# Statistical method for the determination of equivalence of automated test procedures

**DOI:** 10.1155/S146392460300021X

**Published:** 2003

**Authors:** K. Rick Lung, Mary A. Gorko, Jennifer Llewelyn, Norman Wiggins

**Affiliations:** AstraZeneca Pharmaceuticals LP 1800 Concord Pike Wilmington DE 19850 USA

## Abstract

In the development of test methods for solid dosage forms, manual test procedures for assay and content uniformity often precede the development of automated test procedures. Since the mode of extraction for automated test methods is often slightly different from that of the manual test method, additional validation of an automated test method is usually required. In addition to compliance with validation guidelines, developers of automated test methods are often asked to demonstrate equivalence between the manual and automated test methods. There are problems associated with using the traditional zero-difference hypothesis tests (such as the Student's t-test) for demonstrating equivalence. The use of the Westlake Interval and Schuirmann's Two One-sided test as more rigorous methods of demonstrating equivalence is discussed.


Journal of Automated Methods & Management in Chemistry
Vol. 25, No. 6 (November–December 2003) pp. 123–127

Taylor & Francis
Taylor & Francis Group

### Statistical method for the determination of equivalence of automated test procedures†

**K. Rick Lung*, Mary A. Gorko,**
**Jennifer Llewelyn and Norman Wiggins**

*AstraZeneca Pharmaceuticals LP, 1800 Concord Pike, Wilmington, DE 19850, USA*

*In the development of test methods for solid dosage forms, manual test procedures for assay and content uniformity often precede the development of automated test procedures. Since the mode of extraction for automated test methods is often slightly different from that of the manual test method, additional validation of an automated test method is usually required. In addition to compliance with validation guidelines, developers of automated test methods are often asked to demonstrate equivalence between the manual and automated test methods. There are problems associated with using the traditional zero-difference hypothesis tests (such as the Student's* t*-test) for demonstrating equivalence. The use of the Westlake Interval and Schuirmann's Two One-sided test as more rigorous methods of demonstrating equivalence is discussed.*

### Introduction

In the validation of robotic sample preparation methods for assays, method developers generally follow established guidelines for validation. Validation requirements for analytical test methods have been described in the International Conference on Harmonization (ICH) and Food and Drug Administration (FDA) guidelines [1, 2]. Industry-sponsored validation guidelines for automated test methods that closely parallel ICH guidelines have also been published [3]. In addition to following these validation guidelines, it is often necessary for the automation specialist to demonstrate equivalence between a previously established manual method and the automated method. Several of these methods used to determine equivalence are described below.

### *Comparing absolute difference in means*

If it can be assumed that the procedures for manual and automated methods are similar and both methods have comparable accuracy and precision, a simple protocol can be established to compare the average results of the manual method with the automated method.

For example, acceptance criteria are usually stated in an equivalence testing protocol (table 1).

Although the absolute difference between the means might be used as an acceptance criterion for equivalence, such an approach generally does not account for the inherent variability of laboratory data.

For example, although the means of the two sets of assay data in table 2 are within 2% and meet the acceptance criterion for the assay described in table 1, most analytical chemists would not consider the results from methods 1 and 2 to be equivalent. In most cases, the variability of data is as equally important as the difference in means.

### *Student's* t*-test*

A common way in which the results from a new analytical test method are compared with results from another test method is the Student's *t*-test [4]. The *t*-distribution is often used for small samples when the true variance of the population is unknown.

Typically, the following hypotheses are set up in a Student's *t*-test:

$$H_0 : \mu_x = \mu_y$$
$$H_a : \mu_x \neq \mu_y,$$

where $H_0$ and $H_a$ are the null and alternate hypotheses and $\mu_x$ and $\mu_y$ are the population means.

$T$ is calculated as follows:

$$T = \frac{\bar{x} - \bar{y}}{S_p\sqrt{\frac{1}{n_x} + \frac{1}{n_y}}}$$

where $\bar{x}$ and $\bar{y}$ are the sample means and $n_x$ and $n_y$ are the sample sizes.

The pooled standard deviation $S_p$ is calculated from the pooled variance:

$$S_p^2 = \frac{(n_x - 1)S_x^2 + (n_y - 1)S_y^2}{(n_x - 1) + (n_y - 1)}.$$

The null hypothesis, $H_0$: $\mu_x = \mu_y$, is rejected if the absolute value of the calculated $T$ is greater than a critical $t$ for the relevant degrees of freedom (d.f.) and confidence interval ($\alpha$). The d.f. are equal to $n_x + n_y - 2$.

The null hypothesis in the Student's *t*-test ($H_0$: $\mu_x = \mu_y$) is defined so that the hypothesis will be rejected if the means of two sets of results are not equal to (i.e. different from) each other.

From the authors' experience, the Student's *t*-test does not always give results that intuitively make sense. The potential problem of using the *t*-test for evaluating method equivalence can be illustrated by the following examples.

† This paper was initially presented at the ISLAR 2002 Conference and is reproduced here by kind permission of Zymark Corporation.
* To whom correspondence should be addressed.
e-mail: k.rick.lung@astrazeneca.com

*Table 1. Commonly used absolute difference criteria.*

| Validation parameter | Acceptance criteria |
|---|---|
| Equivalence for content uniformity | Absolute value for the difference between the mean of the results from the manual method and the mean of the automated method must not exceed 3% |
| Equivalence for high-performance liquid chromatography assay | Absolute value of the difference between the mean of the results from the manual method and the mean of the automated method must not exceed 2% |

*Table 2. Example of two data sets with significantly different variability.*

| Method 1 | Method 2 |
|---|---|
| 100.1% | 89.5% |
| 100.0% | 89.0% |
| 99.9% | 125.0% |
| Mean = 100.0% | Mean = 101.2% |

*Table 3. Example of two sets of highly precise laboratory data.*

| | Assay results (six replicates) | Mean |
|---|---|---|
| Manual test method | 100.0, 99.9, 100.0, 99.9, 99.9, 100.1 | 100.0 |
| Automated test method | 99.8, 99.8, 99.7, 99.7, 99.8, 99.8 | 99.8 |

*Table 4. Example of two sets of imprecise laboratory data.*

| | Assay results (three replicates) | Mean |
|---|---|---|
| Original test method | 103.1, 100.5, 110.9 | 104.8 |
| Alternate test method | 85.3, 96.0, 98.1 | 93.1 |

In the example shown in table 3, most analysts will qualitatively conclude that the two sets of results are nearly 'the same' and the data are highly precise. However, the calculated $t$ is 5.07, which is greater than the critical $t$ for 95% and 10 d.f. (2.23). The null hypothesis ($H_0$: $\mu_x = \mu_y$) is rejected and the results 'failed' the $t$-test.

In the other set of data (table 4), the calculated $T$ (2.31) is less than the critical $t$ for 95% confidence and 4 d.f. (2.78). The null hypothesis ($H_0$: $\mu_x = \mu_y$) is therefore accepted and the results 'passed' the $t$-test. From these two examples, it can be seen that the $t$-test favours large variability and small sample sizes.

### *Other commonly used statistical tests*

In addition to the $t$-test, the $F$-test is often used to compare the variance between two sets of data. The $F$-test is used to compare two sets of results and to determine if there is any statistical difference in precision:

$$F = \frac{s_1^2}{s_2^2}$$

where $s_1^2$ and $s_2^2$ are the variances of the two sets of data.

Again, the null hypothesis is $H_0$: $s_1^2 = s_2^2$ and the null hypothesis is rejected if one set of results is more precise than the other one. Although the $F$-test might be a good initial test to compare precision, it cannot be used to assess the similarity in accuracy, demonstrating that similarity in precision is not sufficient to show that two methods are equivalent.

For the comparison of more than two sets of data, analysis of variance (ANOVA) is often used. For example, ANOVA is sometimes used to determine if results from three analysts are significantly different. Similar to the $t$-test, ANOVA is used to test the zero-difference hypothesis. Therefore, the test has the same potential problems as the $t$-test when it is used to determine if results from three or more methods are 'the same'.

### *Equivalence tests*

The Student's $t$-test and ANOVA are statistical tests for a 'zero-difference' hypothesis. Therefore, when the $t$-test or ANOVA are used to compare two sets of results, the following statistical question is asked: is it likely that no difference exists between two sets of results?

Therefore, zero-difference hypothesis tests should only be used if one wants to show that results from two methods are different.

If one wants to determine equivalence, a more appropriate statistical question to ask is perhaps: is there an unacceptable difference between two sets of results?

Instead of using a zero-difference hypothesis, the hypotheses for equivalence testing can be expressed as follows:

$$\begin{aligned} &H_0\colon \mu_x - \mu_y \le \theta_L \quad \text{or} \quad \mu_x - \mu_y \ge \theta_U \\ &H_a\colon \theta_L < \mu_x - \mu_y < \theta_U \end{aligned},$$

where $\theta_L$ and $\theta_U$ are predefined as the upper and lower 'acceptable difference' limits for equivalence. When $|\theta_L|$ and $|\theta_U|$ are equal to each other, there are symmetrical limits for the equivalence test.

For example, if the automated and manual methods are defined to be equivalent to each other if the results from both methods are within 2%, the limits are symmetrical and $\theta_L$ and $\theta_U$ are equal to $-2$ and 2%, respectively.

The selection of an 'acceptable difference' is dependent on the variability of the methods, the sample size and the application. For example, suppose there is an automated high-performance liquid chromatography (HPLC) assay and a manual HPLC assay method for tablets, and these two methods have very low variability. The per cent relative standard deviation (%RSD) is much less than 1% and the HPLC assay will be used for product release with a pass-fail limit of 95–105%.

In this situation, a reasonable 'acceptable difference' between two methods might be 2%. On the other hand, if the same HPLC assay is applied to a content uniformity test, a different acceptable difference limit (e.g. 3%) might be more appropriate.

A similar concept of comparing results from two methods was proposed in the proposed USP <1010> chapter 'Analytical data-interpretation and treatment' in the USP Pharmacopeial Forum [5, 6]. More than one equivalence-testing methodology is available. The Westlake Interval and Schuirmann's Two One-sided test are described below.

*Westlake Interval*

The Westlake Interval was first used by Wilfred Westlake, a statistician at the former SmithKline Pharmaceuticals, for the assessment of bioequivalence data [7]. For example, a bioequivalence test is often conducted to compare the systemic bioavailability of a generic solid dosage formulation against those of the innovator. Unlike many efficacy clinical studies that often involve at least hundreds, and sometimes as many as tens of thousands, of patients, bioequivalence studies are conducted with a small number of healthy volunteers. To evaluate bioequivalence, statistical techniques appropriate for smaller sample sizes are used. The Westlake Interval is one of the tests that can be used to determine bioequivalence [8].

The Westlake Interval can also be used to determine equivalence between automated and manual methods (methods 1 and 2). It is an iterative numerical method and is used to test the hypothesis that the two methods are equivalent (i.e. results are within an acceptable difference). If the interval is too wide, the hypothesis of equivalence is rejected. Computation of the Westlake Interval involves an iterative numerical procedure and must be done with a computer. If the calculated Westlake Interval is less than the predefined acceptable difference, it can be concluded that methods 1 and 2 are equivalent.

*Schuirmann's Two One-sided test*

Schuirmann's Two One-sided test is an alternate equivalence test. In it, the upper and lower one-sided $T$ values ($T_L$ and $T_U$) can be calculated from the difference in sample means ($\bar{x} - \bar{y}$), the upper and lower acceptable difference limits $\theta_L$ and $\theta_U$, the sample size of each set of data ($n_x$ and $n_y$) and the pooled standard deviation ($s_p$) as:

$$\mathrm{H}_{01}\colon \mu_x - \mu_y \le \theta_L \quad \text{or} \quad \mathrm{H}_{02}\colon \mu_x - \mu_y \ge \theta_U$$

$$\mathrm{H}_{a1}\colon \mu_x - \mu_y > \theta_L \quad \text{and} \quad \mathrm{H}_{a2}\colon \mu_x - \mu_y < \theta_U$$

$$T_L = \frac{(\bar{x} - \bar{y}) - \theta_L}{S_p\sqrt{\dfrac{1}{n_x} + \dfrac{1}{n_y}}} \qquad T_U = \frac{(\bar{x} - \bar{y}) - \theta_U}{S_p\sqrt{\dfrac{1}{n_x} + \dfrac{1}{n_y}}}$$

$$\mathrm{H}_{01} \text{ is rejected if } |T_L| > t(\alpha, n_x + n_y - 2)$$

$$\mathrm{H}_{02} \text{ is rejected if } |T_U| > t(\alpha, n_x + n_y - 2).$$

Equivalently, results from Schuirmann's Two One-sided test can be expressed in terms of a confidence interval. If the classical ($1-2\alpha$) confidence interval (e.g. if $\alpha = 0.05$, $1-2\alpha = 90\%$) for $\mu_x - \mu_y$ is within the interval ($\theta_L$, $\theta_U$), both $\mathrm{H}_{01}$ and $\mathrm{H}_{02}$ are rejected and methods 1 and 2 are concluded to be equivalent.

Schuirmann's Two One-sided test is different from the Student's *t*-test because the test does not assume there is no difference in the results. Instead, Schuirmann's Two One-sided test expects some acceptable differences ($\theta_L$ and $\theta_U$) in the comparison. If a bias is not expected between two test methods and one wants to show that the distribution of results is within an acceptable difference, Schuirmann's Two One-sided test can be used. Unlike the Student's *t*-test, Schuirmann's Two One-sided test does not favour samples with large standard deviations. Therefore, Schuirmann's Two One-sided test is another appropriate statistical test for determining equivalence.

$$\mu_x - \mu_y < \theta_L \left[\theta_L \underset{u_x - u_y}{\longleftrightarrow} \theta_U\right] \mu_x - \mu_y > \theta_U$$

*Figure 1. Confidence interval from the two one-sided* t*-test.*

### Experimental

All assay and content uniformity results were generated with proprietary tablet formulations. Manual test methods were conducted with typical volumetric glassware and all automated results were obtained from a Zymark TPW-II workstation method. All results were converted to a per cent of the label claim before statistical analyses were conducted.

To include the normal day-to-day variability in the data used for comparison, three sets of TPW-II test results obtained on three separate days were compared with the same number of sets of manual test results. Manual assay determinations were done by three operators on three separate days using independent reference standard solutions. A different HPLC was used for each set of manual assay data.

For automated test results, the same TPW-II was used for all sample preparations. However, independent reference standard solutions were prepared for each set of determinations and HPLC injections were done on three separate HPLCs on different days. For content uni-

*Table 5. High-performance liquid chromatography assay data for tablet strength A.*

| TPW-II test results | | | Manual test results | | |
|---|---|---|---|---|---|
| Set 1 | Set 2 | Set 3 | Set 1 | Set 2 | Set 3 |
| 100.30 | 99.95 | 100.30 | 99.80 | 99.15 | 99.95 |
| 99.20 | 99.75 | 99.95 | 98.95 | 99.30 | 99.30 |
| 99.75 | 100.00 | 100.20 | 99.75 | 98.70 | 99.50 |
| 99.85 | 100.10 | 99.65 | 98.30 | 99.25 | 98.50 |
| 99.70 | 99.65 | 99.55 | 99.90 | 99.20 | 98.85 |
| 100.00 | 99.75 | 101.25 | 97.40 | 99.85 | 99.20 |

*Table 6. High-performance liquid chromatography assay data for tablet strength B.*

| TPW-II test results | | | Manual test results | | |
|---|---|---|---|---|---|
| Set 1 | Set 2 | Set 3 | Set 1 | Set 2 | Set 3 |
| 100.37 | 100.43 | 99.47 | 99.27 | 97.67 | 99.37 |
| 99.90 | 99.10 | 99.63 | 97.97 | 99.73 | 99.30 |
| 99.90 | 99.77 | 99.27 | 98.37 | 99.13 | 98.43 |
| 99.80 | 100.07 | 99.60 | 99.40 | 99.80 | 99.10 |
| 99.30 | 100.30 | 99.47 | 99.10 | 99.43 | 99.07 |
| 99.03 | 99.97 | 98.83 | 99.67 | 98.57 | 99.57 |

*Table 7. Content uniformity data for tablet strength C.*

| TPW-II test results (high-performance liquid chromatography) | | | Manual test results (ultraviolet-visible spectrometry) | | |
|---|---|---|---|---|---|
| Set 1 | Set 2 | Set 3 | Set 1 | Set 2 | Set 3 |
| 101.3 | 100.4 | 97.9 | 98.8 | 100.7 | 101.0 |
| 99.1 | 101.8 | 99.2 | 103.0 | 100.2 | 99.4 |
| 98.7 | 100.4 | 98.2 | 100.5 | 100.0 | 98.6 |
| 98.3 | 99.8 | 98.4 | 102.0 | 100.1 | 96.9 |
| 99.2 | 100.8 | 100.0 | 99.7 | 99.2 | 99.2 |
| 98.7 | 100.5 | 94.9 | 101.4 | 99.8 | 99.7 |
| 99.8 | 99.6 | 98.3 | 100.3 | 99.2 | 101.4 |
| 99.6 | 100.4 | 99.7 | 100.7 | 100.2 | 98.9 |
| 98.8 | 99.1 | 99.6 | 100.1 | 98.8 | 100.5 |
| 99.4 | 98.8 | 98.7 | 101.3 | 100.2 | 101.1 |

formity determinations, the method of detection for the manual method was ultraviolet-visible spectrometry and the method of detection for the automated method was HPLC.

### Results

The percent of label values, Westlake Interval and Schuirmann's Two One-sided test results are presented in tables 5–7.

Manual and automated results from separate days were combined and tested for equivalence using the Westlake Interval and Schuirmann's Two One-sided tests.

### *Westlake Interval results*

For HPLC assay strength A, the mean for the automated results was 99.94% and the mean of the manual results was 99.16%. The 95% Westlake Interval was 1.09%. The calculated Westlake Interval was less than the predefined acceptable difference of 2.0%, and the automated and manual results were equivalent by the Westlake Interval test.

For HPLC assay strength B, the mean for the automated results was 99.68% and the mean of the manual results was 99.00%. The 95% Westlake Interval was 0.99%. Again, the calculated Westlake Interval was less than the predefined acceptable difference of 2.0%, and the automated and manual results were equivalent.

For content uniformity, the mean for the automated results was 99.3% and the mean of the manual results was 100.1%. The 95% Westlake Interval was 1.31%. The calculated Westlake Interval was less than the predefined acceptable difference of 3.0% for content uniformity. Again, the automated and manual results were equivalent.

*Schuirmann's Two One-sided test results*

For Schuirmann's Two One-sided test, an acceptable difference of ±2% will be used for assay results ($\theta_L = -2.0\%$ and $\theta_U = 2.0\%$) and an acceptable difference of ±3% will be used for content uniformity ($\theta_L = -3.0\%$ and $\theta_U = 3.0\%$).

For HPLC assay strength A, the estimated difference between the automated and manual results was 0.78%, the standard error was 0.1836%, and $t(34, 0.05) = 1.69092$. The confidence interval was equal to 0.78% ± 0.1836% (1.69092). Since the calculated confidence interval (0.47–1.09%) was within −2.0 and 2.0%, it was concluded that the assay results for strength A were equivalent.

The estimated difference between the automated and manual results for assay strength B was 0.68%, the standard error was 0.1824%, and $t(34, 0.05) = 1.69092$. The confidence interval was equal to 0.68% ± 0.1824% (1.69092). The calculated confidence interval (0.37–0.99%) was within −2.0 and 2.0%, and it can be concluded that the automated and manual assay results for strength B were equivalent.

For content uniformity strength C, the estimated difference was −0.78%, standard error was 0.315% and $t(58, 0.05) = 1.67155$. The calculated confidence interval (−1.31 to −0.26%) was within ($\theta_L = -3.0\%$ and $\theta_U = 3.0\%$), and the automated and manual results were equivalent.

### Discussion

As shown by the assay and content uniformity examples, the Westlake Interval and Schuirmann's Two One-sided tests are effective and unequivocal statistical methods for evaluating equivalence between automated and manual test results for assay and content uniformity. The Student's *t*-test was shown to be inappropriate for equivalence testing. If the *t*-test had been done on the assay and content uniformity data, all three sets of data would have 'failed' the *t*-test because the test is not designed to demonstrate equivalence.

The use of the confidence interval from Schuirmann's Two One-sided test is consistent with the approach outlined in the proposed USP < 1010 > chapter [5, 6]. However, caution must be used if a statistical equivalence test is used to compare degradation products at trace levels since the uncertainty of measurement at trace level is often much larger. The use of statistical equivalence tests described herein is currently under further evaluation at AstraZeneca.

### Acknowledgements

The authors acknowledge Joe Ycas for pioneering the use of the Westlake Interval in chemistry, manufacturing and control problems.

### References

1. ICH Quality Guidelines Q2A & Q2B, Validation of analytical procedures, in International Conference on Harmonization (ICH) of Technical Requirements for Registration of Pharmaceuticals for Human Use.
2. FDA Guidance Document, *Analytical Procedures and Methods Validation (Draft)* (available at: http://www.fda.gov/cder).
3. Pharmaceutical Analytical Sciences Group (PASG), UK, *Guidance on the Development and Validation of Automated Methods for Finished Product Testing*, vol. 1 (19 July 1999) (available at: http://www.pasg.org.uk).
4. Miller, J. C. and Miller, J. N., *Statistics for Analytical Chemistry*, 3rd edn (New York: Ellis Horwood PTR/Prentice Hall, 1993), p. 55.
5. Pharmacopeial Forum, **27** (2001), 3086.
6. Pharmacopeial Forum, **29** (2003), 194.
7. Westlake, W. J., *Biometrics*, **32** (1976), 741.
8. Chow, S.-C. and Liu, J.-P., *Design and Analysis of Bioavailability and Bioequivalence Studies* (New York: Marcel Dekker, 1992), p. 77.

